# Supplementary material for: Published patterns of spin in biomedical literature: a protocol for a meta-research study
Source: BMJ Open. 2021 Apr 8;11(4):e043784. doi: 10.1136/bmjopen-2020-043784 (PMC8039230; doi:10.1136/bmjopen-2020-043784)
Supplement: Author's manuscript [file bmjopen-2020-043784.draft_revisions.pdf]

BMJ Open is committed to open peer review. As part of this commitment we make the peer review history of every article we publish publicly available.

When an article is published we post the peer reviewers' comments and the authors' responses online. We also post the versions of the paper that were used during peer review. These are the versions that the peer review comments apply to.

The versions of the paper that follow are the versions that were submitted during the peer review process. They are not the versions of record or the final published versions. They should not be cited or distributed as the published version of this manuscript.

BMJ Open is an open access journal and the full, final, typeset and author-corrected version of record of the manuscript is available on our site with no access controls, subscription charges or pay-per-view fees (http://bmjopen.bmj.com).

If you have any questions on BMJ Open's open peer review process please email info.bmjopen@bmj.com

Published patterns of spin in biomedical literature: A protocol for a meta-research study

Journal:	*BMJ Open*
Manuscript ID	bmjopen-2020-043784
Article Type:	Protocol
Date Submitted by the Author:	13-Aug-2020

Complete List of Authors:	Su, Naichuan; Academic Centre for Dentistry Amsterdam,<br>van der Linden, Michiel; Academic Centre for Dentistry Amsterdam, Social Dentistry<br>van der Heijden, Geert; Academic Centre for Dentistry Amsterdam, Social Dentistry<br>Listl, Stefan; Radboud Universiteit, Department of Dentistry – Quality and Safety of Oral Healthcare<br>Schandelmaier, Stefan; McMaster University Faculty of Health Sciences, Department of Clinical Epidemiology and Biostatistics; University Hospital Basel, Intitute for Clinical Epidemiology and Biostatistics<br>Faggion, Clovis M.; University Hospital Munster

Keywords:	ETHICS (see Medical Ethics), EPIDEMIOLOGY, MEDICAL ETHICS

SCHOLARONE™
Manuscripts

BMJ

I, the Submitting Author has the right to grant and does grant on behalf of all authors of the Work (as defined in the below author licence), an exclusive licence and/or a non-exclusive licence for contributions from authors who are: i) UK Crown employees; ii) where BMJ has agreed a CC-BY licence shall apply, and/or iii) in accordance with the terms applicable for US Federal Government officers or employees acting as part of their official duties; on a worldwide, perpetual, irrevocable, royalty-free basis to BMJ Publishing Group Ltd ("BMJ") its licensees and where the relevant Journal is co-owned by BMJ to the co-owners of the Journal, to publish the Work in this journal and any other BMJ products and to exploit all rights, as set out in our licence.

The Submitting Author accepts and understands that any supply made under these terms is made by BMJ to the Submitting Author unless you are acting as an employee on behalf of your employer or a postgraduate student of an affiliated institution which is paying any applicable article publishing charge ("APC") for Open Access articles. Where the Submitting Author wishes to make the Work available on an Open Access basis (and intends to pay the relevant APC), the terms of reuse of such Open Access shall be governed by a Creative Commons licence – details of these licences and which Creative Commons licence will apply to this Work are set out in our licence referred to above.

Other than as permitted in any relevant BMJ Author's Self Archiving Policies, I confirm this Work has not been accepted for publication elsewhere, is not being considered for publication elsewhere and does not duplicate material already published. I confirm all authors consent to publication of this Work and authorise the granting of this licence.

For peer review only

Published patterns of spin in biomedical literature: A protocol for a meta-research study

Naichuan Su,[1] Michiel van der Linden,[1] Geert van der Heijden,[1] Stefan Listl,[2,3] Stefan Schandelmaier,[4,5] Clovis Mariano Faggion Jr[6]

Author affiliations

[1]Department of Social Dentistry, Academic Centre for Dentistry Amsterdam (ACTA), University of Amsterdam and Vrije Universiteit Amsterdam, Amsterdam, the Netherlands

[2]Department of Dentistry – Quality and Safety of Oral Healthcare, Radboud University Medical Center, Radboud University, Nijmegen, the Netherlands

[3]Section for Translational Health Economics, Medical Faculty, Heidelberg University, Heidelberg, Germany

[4]Institute for Clinical Epidemiology and Biostatistics, Department of Clinical Research, University Hospital and University of Basel, Basel, Switzerland

[5]Department of Health Research Methods, Evidence, and Impact, McMaster University, Hamilton, Ontario, Canada

[6]Department of Periodontology and Operative Dentistry, Faculty of Dentistry, University Hospital Münster, Münster, Germany

Corresponding author:

Naichuan Su, Department of Social Dentistry, Academic Centre for Dentistry Amsterdam (ACTA), University of Amsterdam and Vrije Universiteit Amsterdam, Gustav Mahlerlaan 3004, 1081 LA Amsterdam, the Netherlands.

Email: n.su@acta.nl

Keywords: Spin; reporting bias; misinterpretation; prevalence

Word count: 3956

For peer review only - http://bmjopen.bmj.com/site/about/guidelines.xhtml

Abstract

Introduction Spin is defined as reporting practices that distort the interpretation of results and create misleading conclusions by suggesting more favorable results. Such unjustifiable and misleading misrepresentation may negatively influence the development of further studies, clinical practice, and healthcare policies. Spin manifests in various patterns in different sections of publications (titles, abstracts, and main texts). The primary aim of the present study is to identify reported spin patterns and assess the prevalence of spin in general and the prevalence of spin patterns reported in biomedical literature based on previously published systematic reviews and literature reviews on spin.

Methods and analysis PubMed, EMBASE, and SCOPUS will be searched to identify systematic or literature reviews on spin in biomedicine. To improve the comprehensiveness of the search, the snowballing method will be used to broaden the search. The data on spin-related outcomes and characteristics of the included studies will be extracted. The methodological quality of the included studies will be assessed with selective items of the AMSTAR-2 checklist. A new classification scheme for spin patterns will be developed if the classifications of spin patterns identified in the included studies vary. The prevalence of spin and spin patterns will be pooled based on meta-analyses if the classification schemes for spin are comparable across included studies. Otherwise, the prevalence will be described qualitatively. The seriousness of spin patterns will be assessed based on a Delphi consensus study.

Ethics and dissemination This study has been approved by the Academic Centre for Dentistry Amsterdam (ACTA) Ethics Review Committee (2020250). The study will be submitted to a peer-reviewed scientific journal.

Registration Open Science Framework (OSF): osf.io/hzv6e

For peer review only

For peer review only - http://bmjopen.bmj.com/site/about/guidelines.xhtml

Strength and limitations of this study

- While previous systematic or literature reviews on spin concerned the identification of spin patterns in various study designs, this will be the first systematic review focusing on the prevalence of spin patterns and seriousness of the impact of spin patterns on research and clinical practice based on the previously published systematic or literature reviews.
- This study may give insight into which spin patterns occur most frequently and may cause most serious consequences in research and clinical practice, and thus should be given most attention when researchers, clinicians, and policymakers read and write scientific papers.
- Potential limitation may be that the various definitions of spin and the various classification schemes for spin patterns used in the included studies may impact the pooling and interpretation of the results in the present study.

For peer review only - http://bmjopen.bmj.com/site/about/guidelines.xhtml

Introduction

Spin is defined as intentionally or unintentionally inaccurate, unfair, or partial reporting practices that distort the interpretation of research and create misleading conclusions by suggesting more favorable results.[1] Such unjustifiable and misleading misrepresentation may flatter research and make it more attractive for publication and citation and consequently may receive unwarrantably higher impact scores.

Spin is reported in 57% of the published clinical trials on average based on a systematic review on spin.[1] Spin may lead to overpromising and misleading information in transfer of knowledge, introduce misconceptions to researchers, clinicians, and policymakers, and stimulate ineffective or even harmful financial investments from funding organizations and health institutions. Therefore, spin has a negative impact on advancing healthcare practice and population health, adversely influences health policy planning, adds to research waste, decreases the reproducibility of research, hampers the progress of science, and reduces return-of-investment from research.[1-3]

Spin manifests in different sections of publications (titles, abstracts, and main texts) and in various patterns. For example, the title of a publication may claim a beneficial effect of an experimental intervention which is not supported by the reported findings, or the conclusion of a publication is not supported by the reported findings, or the provided recommendations for clinical practice are not in line with the study conclusion. Those spin patterns can be classified into three main forms: misleading reporting, misleading interpretation, and inappropriate extrapolation.[1] The misleading reporting was defined as incomplete or inadequate reporting of the methods, study analysis, study results, or any important information that could be misleading to the readers, such as the selective reporting of or overemphasis on statistically significant second outcomes but ignoring the statistically non-significant primary outcomes. The misleading interpretation was defined as an interpretation of the study results that could be misleading to the readers, such as the conclusion claiming equivalence or comparable effectiveness for non-significant results. The inappropriate extrapolation was defined as an inappropriate generalization of study results, such as the conclusion

For peer review only - http://bmjopen.bmj.com/site/about/guidelines.xhtml

claiming the recommendation to use the treatment in clinical practice in an observational study.[1 4]

Identification of the spin patterns and prevalence of spin patterns in medical publications may give insight into which spin patterns occur most frequently and should be given most attention when researchers, clinicians, and policymakers read and write scientific papers. In addition, knowledge about the relative frequency of [illegible] patterns would be important to inform the planned development of a checklist to help identify common spin patterns.

Multiple systematic reviews or literature reviews investigated spin in primary studies such as randomized controlled trials (RCTs), non-randomized controlled trials, diagnostic accuracy studies, prognostic studies, or systematic reviews performed in different clinical areas.[2 5-9] In addition, a meta-research study on spin was published in 2019, which aimed to identify spin and patterns of spin in the studies on spin.[10] Almost all these previous studies on spin showed that spin was very common across different types of the primary studies, ironically even in the studies on spin.[10] Another meta-research study on spin was published in 2016.[1] It focused on the theory of spin and the overall prevalence of spin in primary studies based on systematic or literature reviews on spin. To date, however, the prevalence of spin patterns in biomedical publications and seriousness of the impact of different spin patterns on research and clinical practice has not been assessed.

The overall aim of the present study is to derive recommendations for prevention of spin in future research. To approach the evaluation of spin from this perspective, we will (1) identify reported spin patterns and assess the prevalence of spin in general and the prevalence of spin patterns reported in biomedical literature based on previously published systematic reviews and literature reviews on spin; (2) if necessary develop a harmonized classification scheme for spin patterns across study designs; and (3) assess the seriousness of the impact of spin patterns on research and clinical practice.

For peer review only - http://bmjopen.bmj.com/site/about/guidelines.xhtml

Methods and analysis

The present protocol has been registered in the Open Science Framework (OSF) and reported in accordance with the Preferred Reporting Items for Systematic Review and Meta-analysis Protocols (PRISMA-P) statement.[11] The present protocol has been approved by Academic Centre for Dentistry Amsterdam (ACTA) Ethics Review Committee (2020250).

Search strategy

Relevant publications will be searched in electronic bibliographic sources, including PUBMED, EMBASE, and SCOPUS. The full search strategies, which have been modified by a senior librarian from Vrije Universiteit Amsterdam, the Netherlands (see acknowledgment), are described in Table 1. Searches will be limited from the last 10 years (from 2010), as from the first publication on the topic spin.[5]

To improve the comprehensiveness of the search and identify potentially eligible studies which are not retrieved from the databases, the snowballing method will be used to broaden the search.[12] That is, we inspect the references that are cited in the studies eligible for inclusion (backward citation searching).[13] Then, we use "see all similar articles" function at PubMed to check the first 20 "similar studies" for each eligible publication retrieved from either the databases or the lists of references of each eligible publication.

Eligibility criteria

We will include publications that satisfy all of the following criteria:

1. They reported systematic or literature reviews on spin. That is, the publications aimed to examine spin for the primary studies in a specific or broad field of medicine. A review will be defined as systematic when authors of the review made clear the intention of performing a systematic review.

2. They assessed spin of primary studies with any study design (e.g. effect of treatment or prevention studies (including randomized and non-randomised trials) and diagnostic and prognostic accuracy studies, economic studies and systematic reviews) in any field of biomedical sciences;

For peer review only - http://bmjopen.bmj.com/site/about/guidelines.xhtml

3. They reported possible spin patterns and prevalence of spin or of spin patterns in the included primary studies.
4. They were published in the English language.

Two reviewers (NS and CMF) will select a sample (10%) of eligible studies to achieve good agreement (at least 80%) on in- and exclusion of publications, and thereafter the remainder selected by one reviewer (NS).[14] Full texts will be obtained for studies that meet the inclusion criteria, and whenever title and abstract provide insufficient information for inclusion.

Data extraction

The characteristics of the included studies and spin-related outcomes will be extracted from each review directly to a standardized form. The standardized form will be developed in Microsoft Excel software.

The information on the characteristics of the included studies include a) publication year and first authors; b) country/continent of the first author; c) number of authors of the review; d) type of review (systematic vs. literature review); e) medical specialty; f) type of primary study evaluated in the review; g) sponsorship of the review; h) conflict of interest of authors of the review; i) sampling methods used in the reviews.

The spin-related outcomes of the included studies include a) in what sections the spin was assessed (e.g. abstracts, methods, results, and conclusions sections of the primary studies); b) spin patterns identified in the reviews including labels, definitions, and classification schemes; c) prevalence of spin (overall and for different spin patterns) based on their original classification schemes.

Two reviewers (NS and CMF) will extract the data from a sample (10%) of eligible studies and achieve good agreement (at least 80%), with the remainder extracted by one reviewer (NS).[14]

Assessment of methodological quality

The updated version of A MeaSurement Tool to Assess systematic Reviews

For peer review only - http://bmjopen.bmj.com/site/about/guidelines.xhtml

(AMSTAR-2), which includes a total of 16 items, is mainly used to assess the methodological quality of systematic reviews that include randomized or non-randomized studies of healthcare interventions.[14] Because there is no validated tool to evaluate the methodological systematic or literature reviews so far, we will apply selective items of the AMSTAR-2 checklist[14] that fit the purpose of our research to assess the methodological quality of the included systematic or literature review on spin. Eight items from the AMSTAR-2 items are applicable and selected in the present study (Table 2).

The response options to each item include “Yes” and “No”. For some items, “Partial yes” is an additional response option.[14] If an included study fully adheres to the item, the answer to the item is judged as “Yes” and this indicates the study has a good methodological quality in the aspect of such item. If an included study partially adheres to the item, the answer to the item is judged as “Partial yes”. If an included study does not adhere to the item or provides no information to rate the item, the answer to the item is judged as “No”.[14] We will not combine the items to create an over score. Instead, the potential impact of each item on the results of the present study will be considered separately.

Two reviewers (NS and CMF) will evaluate the methodological quality of reviews from a sample (10%) of eligible reviews and achieve good agreement (at least 80%), with the remainder extracted by one reviewer (NS).[14]

Reviewers´ training and double-checking for accuracy

The two assessors (NS and CM) will pilot the forms for the data extraction and the methodological quality assessment using a set of 5% of the included reviews.[15] After the piloting round, the results will be discussed and, if necessary, the forms will be refined.

A third reviewer (MvdL) will independently check a random sample (10%) for the different study´s phases (selection, extraction, and quality assessment of the data) for accuracy. Any remaining disagreements will be discussed among all authors to reach a consensus decision.

For peer review only - http://bmjopen.bmj.com/site/about/guidelines.xhtml

Potential development of a comprehensive classification scheme for spin patterns

We anticipate that the patterns of spin identified in the included studies will vary with respect to their labels, definitions, and classification systems used to organize and present them. If the classifications of patterns of spin (and consequently overall spin which is the sum of different patterns) vary only slightly across reviews, we might be able to directly extract and summarize the reported prevalences (both for overall spin and different spin patterns) across reviews. If, however, the categories of spin patterns (and thus the definitions of overall spin) vary substantially across reviews, a more comprehensive classification scheme will be developed for the spin patterns identified from the previous reviews based on the consensus discussion in the team. Therefore, we will arrange the different patterns as reported, i.e. extract verbatim quotes of labels and definitions, including potentially classification schemes, in a list stratified by reviews. The team members will consider the different definitions and classifications and suggest whether the categories could be harmonized by lumping together and splitting up. If splitting up is necessary, we would have to re-assess the prevalence of patterns of spin in primary studies classified in categories that require splitting up. Therefore, we may have to approach the investigators of the original studies. If the variation of patterns is too large to be easily combined based on the judgment of the team, we may decide to perform a formal consensus study to develop a new classification, involving experts in the field (we would combine this consensus study with the assessment of the seriousness of the impact of spin patterns, see the section "Assessment of the seriousness of the impact of spin patterns"). Then, if possible, the prevalence of each spin pattern in the harmonized classification scheme will be calculated.

Statistical analysis

The overall prevalence of spin presented in the included systematic or literature reviews will be described. If the included reviews used similar classification schemes for spin, the prevalence of spin will be pooled based on meta-analysis for single proportions with random effect models. Otherwise, the prevalence of spin and spin patterns will be described without quantitative analysis. When the meta-analysis is possibly performed, statistical heterogeneity of the meta-analysis will be assessed

For peer review only - http://bmjopen.bmj.com/site/about/guidelines.xhtml

with I^2 test.[16] The heterogeneity is considered large if I^2 >50%. If it shows large heterogeneity and the number of included studies in a meta-analysis is >10, multivariate meta-regression analysis will be used to explore the possible factors for the heterogeneity. To assess the publication bias of the included studies, a funnel plot will be used when the number of the studies included in the meta-analysis is larger than 10.[16] The statistical analysis will be performed via R software 3.3 ((R Development Core Team).

Assessment of the seriousness of the impact of spin patterns

The seriousness of the impact of spin patterns indicates how much consequence a certain spin pattern may cause in research and clinical practice if such spin pattern occurs in biomedical publications. We are planning to perform a Delphi consensus study to rate the seriousness of the spin patterns. Therefore, we will invite experts in the field defined as authors of the included reviews. We will invite one author per included review, or two if the number of reviews is too small. We attempt to include a total of 10-15 experts. If the number of reviews is greater than 15, we will draw a random sample. We will set up an initial video conference to clarify the aim of the consensus study. For the consensus study, we will ask the experts to rate the seriousness of the impact of spin patterns on research and clinical practice using a 7-point rating scale in which "1" indicates that the spin pattern is not serious at all and "7" indicates that the spin pattern is extremely serious. In addition, and most importantly, the experts will also provide a written justification for the rating. The mean scores will be calculated and the range of the scores will be presented for each spin pattern. A researcher from the core team will present the results (i.e. anonymous ratings, justifications, and mean scores) to the individual group members in written form, ask them to review the results, and possibly adjust their own judgments based on the summary. We will repeat this process until no further changes occur. The final version will provide, for each spin pattern, the individual ratings (to show the level of agreement), the mean rating, and a justification (if the ratings are heterogeneous, then we will provide explanation for the disagreement). It is not the aim that all members agree on a specific numerical rating and justification, i.e. disagreement regarding the importance of some pattern is a possible result of the study. If necessary, e.g., to discuss questions that are too complex for written communication, we may set up

For peer review only - http://bmjopen.bmj.com/site/about/guidelines.xhtml

additional video conferences. If necessary, we may first develop a new classification for spin patterns together with the experts using similar methods (see the section "Potential development of a comprehensive classification scheme for spin patterns").

Ethics and dissemination

The present protocol has been approved by Academic Centre for Dentistry Amsterdam (ACTA) Ethics Review Committee (2020250). We will publish results in a peer-reviewed scientific journal.

Authors` contributions

NS and CM drafted the first version of the protocol and subsequently incorporated the suggested revisions. MvdL, GvdH, SL, and SS commented and revised each section of the protocol.

Funding

This research received no specific grant from any funding agency in the public, commercial or not-for-profit sectors.

Competing interests

None declared

Acknowledgement

We gratefully acknowledged Mr. René H.J. Otten, from University Library, Vrije Universiteit Amsterdam, the Netherlands for his assistance in the modification of the search strategies.

Patient consent

Not applicable

Provenance and peer review

Not commissioned; externally peer review

For peer review only - http://bmjopen.bmj.com/site/about/guidelines.xhtml

Patient and public involvement

Patients or the public will not be involved in the design, or conduct, or reporting, or dissemination plans of our research.

References

1. Chiu K, Grundy Q, Bero L. "Spin" in published biomedical literature: A methodological systematic review. *PLoS Biol* 2017;15:e2002173.
2. Ochodo EA, de Haan MC, Reitsma JB, et al. Overinterpretation and misreporting of diagnostic accuracy studies: Evidence of "spin". *Radiology* 2013;267:581-8.
3. Caulfield T, Ogbogu U. The commercialization of university-based research: balancing risks and benefits. *BMC Med Ethics* 2015;16:70.
4. Yavchitz A, Ravaud P, Altman DG, et al. A new classification of spin in systematic reviews and meta-analyses was developed and ranked according to the severity. *J Clin Epidemiol* 2016;75:56-65.
5. Boutron I, Dutton S, Ravaud P, et al. Reporting and interpretation of randomized controlled trials with statistically nonsignificant results for primary outcomes. *JAMA* 2010;303:2058-64.
6. Cooper CM, Gray HM, Ross AE, et al. Evaluation of spin in the abstracts of otolaryngology randomized controlled trials. *Laryngoscope* 2018;21:2036-40.
7. Kempf E, de Beyer JA, Cook J, et al. Overinterpretation and misreporting of prognostic factor studies in oncology: a systematic review. *Br J Cancer* 2018;119:1288-96.
8. Lazarus C, Haneef R, Ravaud P, et al. Classification and prevalence of spin in abstracts of non-randomized studies evaluating and intervention. *BMC Med Res Methodol* 2015;15:85.
9. Boutron I, Altman DG, Hopewell S, et al. Impact of spin in the abstracts of articles reporting results of randomized controlled trials in the field of cancer: the SPIIN randomized controlled trial. *J Clin Oncol* 2014;32:4120-6.
10. Bero L, Chiu K, Grundy Q. The SSSPIN study-spin in studies of spin: meta-research analysis. *BMJ* 2019;367:I6202.
11. Shamseer L, Moher D, Clarke M, et al. Preferred reporting items for systematic review and meta-analysis protocols (PRISMA-P) 2015: elaboration and explanation. *BMJ* 2015;350:g7647.

For peer review only - http://bmjopen.bmj.com/site/about/guidelines.xhtml

12. Liberati A, Altman DG, Tetzlaff J, et al. The PRISMA statement for reporting systematic reviews and meta-analyses of studies that evaluate health care interventions: explanation and elaboration. *PLoS Med* 2009;6:e1000100.
13. Briscoe S, Bethel A, Rogers M. Conduct and reporting of citation searching in Cochrane systematic reviews: A cross-sectional study. *Res Synth Methods* 2020;11:169-80.
14. Shea BJ, Reeves BC, Wells G, et al. AMSTAR 2: a critical appraisal tool for systematic reviews that include randomised or non-randomised studies of healthcare interventions, or both. *BMJ* 2017;358:j4008.
15. Pieper D, Pollock M, Fernandes RM, et al. Epidemiology and reporting characteristics of overviews of reviews of healthcare interventions published 2012-2016: Protocol for a systematic review. *Syst Rev* 2017;6:73.
16. Higgins JPT, Green S, eds. *Cochrane Handbook for Systematic Reviews of Interventions*. *Version 5.1.0. Updated,* 2011.

For peer review only

For peer review only - http://bmjopen.bmj.com/site/about/guidelines.xhtml

Table 1 Search strategies in PubMed, EMBASE, and SCOPUS

**PubMed**	
#11	Search: #10 AND ("2010"[Date-Publication]: "3000"[Date-Publication])
#10	Search: #8 OR #9
#9	Search: #7 AND systematic[sb]
#8	Search: #7 AND review[Publication Type]
#7	Search: #3 OR #4 OR #5 OR #6
#6	Search: ("misinterpretation"[tiab] OR "overinterpretation"[tiab]) AND "result*"[tiab]
#5	Search: "distorted results"[tiab] OR "distorted reporting"[tiab] OR "distorted presentation"[tiab] OR "distorted interpretation"[tiab]
#4	Search: "reporting bias"[tiab] OR "interpretation bias"[tiab]
#3	Search: #1 AND #2
#2	Search: "Publication Bias"[Mesh] OR "report*"[tiab] OR "bias*"[tiab] OR "publish*"[tiab] OR "non-significant*"[tiab] OR "nonsignificant*"[tiab]
#1	Search: "Spin"[tiab]
**EMBASE**	
#11	#10 AND [2010-2020]/py
#10	#8 OR #9
#9	#7 AND 'systematic review*':ti,ab,kw
#8	#7 AND [review]/lim
#7	#3 OR #4 OR #5 OR #6
#6	(('misinterpretation' OR 'overinterpretation') NEAR/4 'result*'):ti,ab,kw
#5	distorted results':ti,ab,kw OR 'distorted reporting':ti,ab,kw OR 'distorted presentation':ti,ab,kw OR 'distorted interpretation':ti,ab,kw
#4	'reporting bias'/exp OR 'interpretation bias'/exp
#3	#1 AND #2
#2	'publication bias'/exp OR 'report*':ti,ab,kw OR 'bias*':ti,ab,kw OR 'publish*':ti,ab,kw OR 'non-significant*':ti,ab,kw OR 'nonsignificant*':ti,ab,kw
#1	'spin':ti,ab,kw

For peer review only - http://bmjopen.bmj.com/site/about/guidelines.xhtml

SCOPUS	
#10	#9 AND (PUBYEAR > 2009)
#9	#6 OR #8
#8	#4 AND #7
#7	TITLE-ABS-KEY ("systematic review*")
#6	#4 OR #5
#5	DOCTYPE (re)
#4	#1 OR #2 OR #3
#3	TITLE-ABS-KEY (("misinterpretation" OR "overinterpretation") W/3 ("result*"))
#2	TITLE-ABS-KEY ("reporting bias" OR "interpretation bias" OR "distorted results" OR "distorted reporting" OR "distorted presentation " OR "distorted interpretation")
#1	TITLE-ABS-KEY (("spin") W/3 ("report*" OR "bias*" OR "publish*" OR "non-significant*" OR "nonsignificant*"))

For peer review only

Table 2 The critical and non-critical items of AMSTAR-2 that are applicable in the present study

Items	Response options
Critical items	
Item 4 Did the review authors use a comprehensive literature search strategy?	Yes/Partial yes/No
Item 7 Did the review authors provide a list of excluded studies and justify the exclusions?	Yes/Partial yes/No
Item 13 Did the review authors account for RoB in individual studies when interpreting/discussing the results of the review?	Yes/No
Non-critical items	
Item 5 Did the review authors perform study selection in duplicate?	Yes/No
Item 6 Did the review authors perform data extraction in duplicate?	Yes/No
Item 8 Did the review authors describe the included studies in adequate detail?	Yes/Partial yes/No
Item 10 Did the review authors report on the sources of funding for the studies included in the review?	Yes/No
Item 16 Did the review authors report any potential sources of conflict of interest, including any funding they received for conducting the review?	Yes/No

For peer review only

PRISMA-P 2015 Checklist

This checklist has been adapted for use with protocol submissions to *Systematic Reviews* from Table 3 in Moher D et al: Preferred reporting items for systematic review and meta-analysis protocols (PRISMA-P) 2015 statement. *Systematic Reviews* 2015 **4**:1

Section/topic	#	Checklist item	Information reported Yes	Information reported No	Page number(s)
**ADMINISTRATIVE INFORMATION**					
**Title**					
Identification	1a	Identify the report as a protocol of a systematic review	✓	☐	1
Update	1b	If the protocol is for an update of a previous systematic review, identify as such	☐	☐	N.A.
**Registration**	2	If registered, provide the name of the registry (e.g., PROSPERO) and registration number in the Abstract	✓	☐	2
**Authors**					
Contact	3a	Provide name, institutional affiliation, and e-mail address of all protocol authors; provide physical mailing address of corresponding author	✓	☐	1
Contributions	3b	Describe contributions of protocol authors and identify the guarantor of the review	✓	☐	11
**Amendments**	4	If the protocol represents an amendment of a previously completed or published protocol, identify as such and list changes; otherwise, state plan for documenting important protocol amendments	☐	☐	N.A.
**Support**					
Sources	5a	Indicate sources of financial or other support for the review	✓	☐	11
Sponsor	5b	Provide name for the review funder and/or sponsor	☐	☐	N.A.
Role of sponsor/funder	5c	Describe roles of funder(s), sponsor(s), and/or institution(s), if any, in developing the protocol	☐	☐	N.A.
**INTRODUCTION**					
**Rationale**	6	Describe the rationale for the review in the context of what is already known	✓	☐	4-5
**Objectives**	7	Provide an explicit statement of the question(s) the review will address with reference to participants, interventions, comparators, and outcomes (PICO)	✓	☐	5

BioMed Central
The Open Access Publisher

Section/topic	#	Checklist item	Information reported: Yes	Information reported: No	Page number(s)
**METHODS**					
**Eligibility criteria**	8	Specify the study characteristics (e.g., PICO, study design, setting, time frame) and report characteristics (e.g., years considered, language, publication status) to be used as criteria for eligibility for the review	✓	☐	6-7
**Information sources**	9	Describe all intended information sources (e.g., electronic databases, contact with study authors, trial registers, or other grey literature sources) with planned dates of coverage	✓	☐	6
**Search strategy**	10	Present draft of search strategy to be used for at least one electronic database, including planned limits, such that it could be repeated	✓	☐	14-15 (Table 1)
STUDY RECORDS					
Data management	11a	Describe the mechanism(s) that will be used to manage records and data throughout the review	✓	☐	7
Selection process	11b	State the process that will be used for selecting studies (e.g., two independent reviewers) through each phase of the review (i.e., screening, eligibility, and inclusion in meta-analysis)	✓	☐	7-9
Data collection process	11c	Describe planned method of extracting data from reports (e.g., piloting forms, done independently, in duplicate), any processes for obtaining and confirming data from investigators	✓	☐	7
**Data items**	12	List and define all variables for which data will be sought (e.g., PICO items, funding sources), any pre-planned data assumptions and simplifications	✓	☐	7
**Outcomes and prioritization**	13	List and define all outcomes for which data will be sought, including prioritization of main and additional outcomes, with rationale	✓	☐	7
**Risk of bias in individual studies**	14	Describe anticipated methods for assessing risk of bias of individual studies, including whether this will be done at the outcome or study level, or both; state how this information will be used in data synthesis	✓	☐	7-8
DATA					
**Synthesis**	15a	Describe criteria under which study data will be quantitatively synthesized	✓	☐	10
	15b	If data are appropriate for quantitative synthesis, describe planned summary measures, methods of handling data, and methods of combining data from studies, including any planned exploration of consistency (e.g., I^2, Kendall's tau)	✓	☐	10
	15c	Describe any proposed additional analyses (e.g., sensitivity or subgroup analyses, meta-regression)	✓	☐	10
	15d	If quantitative synthesis is not appropriate, describe the type of summary planned	✓	☐	10
**Meta-bias(es)**	16	Specify any planned assessment of meta-bias(es) (e.g., publication bias across studies, selective reporting within studies)	✓	☐	10
**Confidence in**	17	Describe how the strength of the body of evidence will be assessed (e.g., GRADE)	☐	☐	N.A.

For peer review only

Section/topic	#	Checklist item	Information reported		Page number(s)
			Yes	No	
**cumulative evidence**					

For peer review only

Published patterns of spin in biomedical literature: A protocol for a meta-research study

Journal:	*BMJ Open*
Manuscript ID	bmjopen-2020-043784.R1
Article Type:	Protocol
Date Submitted by the Author:	07-Feb-2021
Complete List of Authors:	Su, Naichuan; Academic Centre for Dentistry Amsterdam,<br>van der Linden, Michiel; Academic Centre for Dentistry Amsterdam, Social Dentistry<br>van der Heijden, Geert; Academic Centre for Dentistry Amsterdam, Social Dentistry<br>Listl, Stefan; Radboud Universiteit, Department of Dentistry – Quality and Safety of Oral Healthcare<br>Schandelmaier, Stefan; McMaster University Faculty of Health Sciences, Department of Clinical Epidemiology and Biostatistics; University Hospital Basel, Intitute for Clinical Epidemiology and Biostatistics<br>Faggion, Clovis M.; University Hospital Munster
**Primary Subject Heading**:	Ethics
Secondary Subject Heading:	Epidemiology, Evidence based practice, Health policy
Keywords:	ETHICS (see Medical Ethics), EPIDEMIOLOGY, MEDICAL ETHICS

SCHOLARONE™
Manuscripts

I, the Submitting Author has the right to grant and does grant on behalf of all authors of the Work (as defined in the below author licence), an exclusive licence and/or a non-exclusive licence for contributions from authors who are: i) UK Crown employees; ii) where BMJ has agreed a CC-BY licence shall apply, and/or iii) in accordance with the terms applicable for US Federal Government officers or employees acting as part of their official duties; on a worldwide, perpetual, irrevocable, royalty-free basis to BMJ Publishing Group Ltd ("BMJ") its licensees and where the relevant Journal is co-owned by BMJ to the co-owners of the Journal, to publish the Work in this journal and any other BMJ products and to exploit all rights, as set out in our licence.

The Submitting Author accepts and understands that any supply made under these terms is made by BMJ to the Submitting Author unless you are acting as an employee on behalf of your employer or a postgraduate student of an affiliated institution which is paying any applicable article publishing charge ("APC") for Open Access articles. Where the Submitting Author wishes to make the Work available on an Open Access basis (and intends to pay the relevant APC), the terms of reuse of such Open Access shall be governed by a Creative Commons licence – details of these licences and which Creative Commons licence will apply to this Work are set out in our licence referred to above.

Other than as permitted in any relevant BMJ Author's Self Archiving Policies, I confirm this Work has not been accepted for publication elsewhere, is not being considered for publication elsewhere and does not duplicate material already published. I confirm all authors consent to publication of this Work and authorise the granting of this licence.

For peer review only

Published patterns of spin in biomedical literature: A protocol for a meta-research study

Naichuan Su,[1] Michiel van der Linden,[1] Geert van der Heijden,[1] Stefan Listl,[2,3] Stefan Schandelmaier,[4,5] Clovis Mariano Faggion Jr[6]

Author affiliations

[1]Department of Social Dentistry, Academic Centre for Dentistry Amsterdam (ACTA), University of Amsterdam and Vrije Universiteit Amsterdam, Amsterdam, the Netherlands

[2]Department of Dentistry – Quality and Safety of Oral Healthcare, Radboud University Medical Center, Radboud University, Nijmegen, the Netherlands

[3]Section for Translational Health Economics, Medical Faculty, Heidelberg University, Heidelberg, Germany

[4]Institute for Clinical Epidemiology and Biostatistics, Department of Clinical Research, University Hospital and University of Basel, Basel, Switzerland

[5]Department of Health Research Methods, Evidence, and Impact, McMaster University, Hamilton, Ontario, Canada

[6]Department of Periodontology and Operative Dentistry, Faculty of Dentistry, University Hospital Münster, Münster, Germany

Corresponding author:

Naichuan Su, Department of Social Dentistry, Academic Centre for Dentistry Amsterdam (ACTA), University of Amsterdam and Vrije Universiteit Amsterdam, Gustav Mahlerlaan 3004, 1081 LA Amsterdam, the Netherlands.

Email: n.su@acta.nl

Keywords: Spin; reporting bias; misinterpretation; prevalence

Word count: 4276

For peer review only - http://bmjopen.bmj.com/site/about/guidelines.xhtml

Abstract

Introduction Spin is defined as reporting practices that distort the interpretation of results and create misleading conclusions by suggesting more favorable results. Such unjustifiable and misleading misrepresentation may negatively influence the development of further studies, clinical practice, and healthcare policies. Spin manifests in various patterns in different sections of publications (titles, abstracts, and main texts). The primary aim of the present study is to identify reported spin patterns and assess the prevalence of spin in general and the prevalence of spin patterns reported in biomedical literature based on previously published systematic reviews and literature reviews on spin.

Methods and analysis PubMed, EMBASE, and SCOPUS will be searched to identify systematic or literature reviews on spin in biomedicine. To improve the comprehensiveness of the search, the snowballing method will be used to broaden the search. The data on spin-related outcomes and characteristics of the included studies will be extracted. The methodological quality of the included studies will be assessed with selective items of the AMSTAR-2 checklist. A new classification scheme for spin patterns will be developed if the classifications of spin patterns identified in the included studies vary. The prevalence of spin and spin patterns will be pooled based on meta-analyses if the classification schemes for spin are comparable across included studies. Otherwise, the prevalence will be described qualitatively. The seriousness of spin patterns will be assessed based on a Delphi consensus study.

Ethics and dissemination This study has been approved by the Academic Centre for Dentistry Amsterdam (ACTA) Ethics Review Committee (2020250). The study will be submitted to a peer-reviewed scientific journal.

Registration Open Science Framework (OSF): osf.io/hzv6e

For peer review only - http://bmjopen.bmj.com/site/about/guidelines.xhtml

Strength and limitations of this study

- While previous systematic or literature reviews on spin concerned the identification of spin patterns in various study designs, this will be the first systematic review focusing on the prevalence of spin patterns and seriousness of the impact of spin patterns on research and clinical practice based on the previously published systematic or literature reviews.
- This study may give insight into which spin patterns occur most frequently and may cause most serious consequences in research and clinical practice, and thus should be given most attention when researchers, clinicians, and policymakers read and write scientific papers.
- Potential limitation may be that the various definitions of spin and the various classification schemes for spin patterns used in the included studies may impact the pooling and interpretation of the results in the present study.

For peer review only

Introduction

Spin is defined as intentionally or unintentionally inaccurate, unfair, or partial reporting practices that distort the interpretation of research and create misleading conclusions by suggesting more favorable results.[1] It can be hypothesized that such unjustifiable and misleading misrepresentation may flatter research and make it more attractive for publication and citation and consequently may receive unwarrantably higher impact scores.

Spin is reported in 57% of the published clinical trials on average based on a systematic review on spin.[1] Based on the hypothesis, spin may lead to overpromising and misleading information in transfer of knowledge, introduce misconceptions to researchers, clinicians, and policymakers, and stimulate ineffective or even harmful financial investments from funding organizations and health institutions. Therefore, spin has a negative impact on advancing healthcare practice and population health, adversely influences health policy planning, adds to research waste, decreases the reproducibility of research, hampers the progress of science, and reduces return-of-investment from research.[1-3]

Spin manifests in different sections of publications (titles, abstracts, and main texts) and in various patterns. For example, the title of a publication may claim a beneficial effect of an experimental intervention which is not supported by the reported findings, or the conclusion of a publication is not supported by the reported findings, or the provided recommendations for clinical practice are not in line with the study conclusion. Those spin patterns can be classified into three main forms: misleading reporting, misleading interpretation, and inappropriate extrapolation.[4 5] The misleading reporting was defined as incomplete or inadequate reporting of the methods, study analysis, study results, or any important information that could be misleading to the readers, such as the selective reporting of or overemphasis on statistically significant secondary outcomes but ignoring the statistically non-significant primary outcomes. The misleading interpretation was defined as an interpretation of the study results that could be misleading to the readers, such as the conclusion claiming equivalence or comparable effectiveness for non-significant

For peer review only - http://bmjopen.bmj.com/site/about/guidelines.xhtml

results. The inappropriate extrapolation was defined as an inappropriate generalization of study results, such as the conclusion claiming the recommendation to use the treatment in clinical practice in an observational study.[1 4]

Several studies on the prevalence of spin and the impact of spin on research in general have been published. For example, multiple systematic reviews or literature reviews investigated spin in primary studies such as randomized controlled trials (RCTs), non-randomized controlled trials, diagnostic accuracy studies, prognostic studies, or systematic reviews performed in different clinical areas.[2 5-8] In addition, a meta-research study on spin was published in 2019, which aimed to identify spin and patterns of spin in the studies on spin.[10] Almost all these previous studies on spin showed that spin was very common across different types of the primary studies, ironically even in the studies on spin.[10] In addition, a study reported that spin in abstracts may influence clinicians` decision-making on the treatment effect in the field of cancer,[9] while another study reported that spin in health news stories reporting studies of pharmacologic treatment may influence both patients` and caregivers` decision-making on the treatment effect.[11] Another meta-research study on spin was published in 2016.[1] It focused on the theory of spin and the overall prevalence of spin in primary studies based on systematic or literature reviews on spin. These studies have revealed that spin is highly frequent in medical publications and can significantly impact both clinicians` and patients` decision-making in clinical practice. The main aim of these studies was to define, describe and understand spin. From the publications on spin available to date, different patterns of spin seem to emerge, but a comprehensive overview of spin patterns is lacking. Spin patterns vary in the prevalence in research and in the consequences on research. The ignorance on those spin patterns with the highest prevalence and the most serious impact on research may widely and severely harm the reliability, transparency, and accuracy of translation, dissemination, and implementation of the evidence from medical and health research to practice. Identification of the prevalence and the impact of spin patterns will help increase the awareness of researchers, clinicians, reviewers, and policymakers on which spin patterns should be given most attention when they read, review and write scientific papers. Besides, to develop the practical and targeted guidance on the identification and prevention of spin in research, the manifestations

For peer review only - http://bmjopen.bmj.com/site/about/guidelines.xhtml

of spin should be known. The specific and targeted guidance on how to identify and prevent such spin patterns in research can be made accordingly to help prevent spin in the future publications and prevent the use and transfer of the spinned evidence in research. To date, however, meta-research studies on the prevalence of spin patterns in biomedical publications and seriousness of the impact of different spin patterns on research and clinical practice are scarce.

Therefore, the aims of the study are to (1) identify reported spin patterns and assess the prevalence of spin in general and the prevalence of spin patterns reported in biomedical literature based on previously published systematic reviews and literature reviews on spin; and (2) assess the seriousness of the impact of spin patterns on research and clinical practice. Based on the spin patterns found in the current study which are most frequent in research or have the most serious consequences on research, we will derive recommendations for researchers, clinicians, and policymakers to prevent such spin patterns in future research.

Methods and analysis

The present protocol has been registered in the Open Science Framework (OSF) and reported in accordance with the Preferred Reporting Items for Systematic Review and Meta-analysis Protocols (PRISMA-P) statement.[12] The present protocol has been approved by Academic Centre for Dentistry Amsterdam (ACTA) Ethics Review Committee (2020250).

Search strategy

Relevant publications will be searched in electronic bibliographic sources, including PUBMED, EMBASE, and SCOPUS. The full search strategies, which have been modified by a senior librarian from Vrije Universiteit Amsterdam, the Netherlands (see acknowledgment), are described in Table 1. Searches will be limited from the last 10 years (from 2010), as from the first publication to identify spin in clinical trials.[6]

To improve the comprehensiveness of the search and identify potentially eligible studies which are not retrieved from the databases, the snowballing method will be

For peer review only - http://bmjopen.bmj.com/site/about/guidelines.xhtml

used to broaden the search.[13] That is, we inspect the references that are cited in the studies eligible for inclusion (backward citation searching).[14] Then, we use "see all similar articles" function at PubMed to check the first 20 most "similar studies" for each eligible publication retrieved from either the databases or the lists of references of each eligible publication.

Eligibility criteria

We will include publications that satisfy all of the following criteria:

1. They reported systematic or literature reviews on spin. That is, the publications aimed to examine spin for the primary studies in a specific or broad field of medicine. The review articles are defined as a more or less systematic way of collecting and synthesizing previous research.[15] A review will be defined as systematic when authors of the review made clear the intention of performing a systematic review. If a publication met the definition based on its adopted methods without using the word "review" in the titles, abstracts, or main texts, it will also be included.
2. They assessed spin of primary studies with any study design (e.g. effect of treatment or prevention studies (including randomized and non-randomised trials) and diagnostic and prognostic accuracy studies, economic studies and systematic reviews) in any field of biomedical sciences;
3. They reported possible spin patterns and prevalence of spin or of spin patterns in the included primary studies.
4. They were published in the English language.

Two reviewers (NS and CMF) will select a sample (10%) of eligible studies to achieve good agreement (at least 80%) on in- and exclusion of publications, and thereafter the remainder selected by one reviewer (NS).[16] Full texts will be obtained for studies that meet the inclusion criteria, and whenever title and abstract provide insufficient information for inclusion.

Data extraction

The characteristics of the included studies and spin-related outcomes will be extracted from each review directly to a standardized form. The standardized form will be

For peer review only - http://bmjopen.bmj.com/site/about/guidelines.xhtml

developed in Microsoft Excel software.

The information on the characteristics of the included studies include a) publication year and first authors; b) country/continent of the first author; c) number of authors of the review; d) type of review (systematic vs. literature review); e) medical specialty; f) type of primary study evaluated in the review; g) sponsorship of the review; h) conflict of interest of authors of the review; i) sampling methods used in the reviews.

The spin-related outcomes of the included studies include a) in what sections the spin was assessed (e.g. abstracts, methods, results, and conclusions sections of the primary studies); b) spin patterns identified in the reviews including labels, definitions, and classification schemes; c) prevalence of spin (overall and for different spin patterns) based on their original classification schemes.

Two reviewers (NS and CMF) will extract the data from a sample (10%) of eligible studies and achieve good agreement (at least 80%), with the remainder extracted by one reviewer (NS).[16]

Assessment of methodological quality

The updated version of A MeaSurement Tool to Assess systematic Reviews (AMSTAR-2), which includes a total of 16 items, is mainly used to assess the methodological quality of systematic reviews that include randomized or non-randomized studies of healthcare interventions.[16] Because there is no validated tool to evaluate the methodological systematic or literature reviews so far, we will apply selective items of the AMSTAR-2 checklist[16] that fit the purpose of our research to assess the methodological quality of the included systematic or literature review on spin. Eight items from the AMSTAR-2 items are applicable and selected in the present study (Table 2).

The response options to each item include “Yes” and “No”. For some items, “Partial yes” is an additional response option.[16] If an included study fully adheres to the item, the answer to the item is judged as “Yes” and this indicates the study has a good methodological quality in the aspect of such item. If an included study partially

For peer review only - http://bmjopen.bmj.com/site/about/guidelines.xhtml

adheres to the item, the answer to the item is judged as “Partial yes”. If an included study does not adhere to the item or provides no information to rate the item, the answer to the item is judged as “No”.[16] We will not combine the items to create an overall score. Instead, the potential impact of each item on the results of the present study will be considered separately.

Two reviewers (NS and CMF) will evaluate the methodological quality of reviews from a sample (10%) of eligible reviews and achieve good agreement (at least 80%), with the remainder extracted by one reviewer (NS).[16]

Reviewers´ training and double-checking for accuracy

The two assessors (NS and CMF) will pilot the forms for the data extraction and the methodological quality assessment using a set of 5% of the included reviews.[17] After the piloting round, the results will be discussed and, if necessary, the forms will be refined.

A third reviewer (MvdL) will independently check a random sample (10%) for the different study´s phases (selection, extraction, and quality assessment of the data) for accuracy. Any remaining disagreements will be discussed among all authors to reach a consensus decision.

Statistical analysis

The overall prevalence of spin and the prevalence of each spin pattern presented in the included systematic or literature reviews will be described. We anticipate that the spin patterns identified in the included studies will vary with respect to their labels, definitions, and classification systems used to organize and present them. If the classifications of patterns of spin (and consequently overall spin which is the sum of different patterns) vary only slightly across reviews, we will pool the reported overall prevalences and the prevalences of each spin pattern separately across reviews based on meta-analysis for single proportions with random effect models. If, however, the categories of spin patterns (and thus the definitions of overall spin) vary substantially across reviews, the overall prevalence of spin and the prevalence of spin patterns will be described qualitatively. When the meta-analysis is possibly performed, statistical

For peer review only - http://bmjopen.bmj.com/site/about/guidelines.xhtml

heterogeneity of the meta-analysis will be assessed with I^2 test.[18] The heterogeneity is considered large if I^2 >50%. If it shows large heterogeneity and the number of included studies in a meta-analysis is >10, multivariate meta-regression analysis will be used to explore the possible factors for the heterogeneity. To assess the publication bias of the included studies, a funnel plot will be used when the number of the studies included in the meta-analysis is larger than 10.[18] The statistical analysis will be performed via R software 3.3 ((R Development Core Team).

Assessment of the seriousness of the impact of spin patterns

The seriousness of the impact of spin patterns indicates how much consequence a certain spin pattern may cause in research and clinical practice if such spin pattern occurs in biomedical publications. We are planning to perform a Delphi consensus study to rate the seriousness of the spin patterns. The Delphi method is a structured process which uses a series of questionnaires or rounds to gather and to provide information in a panel of experts.[19] Compared with other possible study designs (e.g. RCTs), the Delphi method is more simple to design and more flexible to conduct.[20] Besides, it enables anonymity, which encourages experts` creativity, honesty, independent thinking, and balanced consideration of ideas while reducing the risk of group dynamics negatively affecting the outcomes.[20 21] The experts` opinions are given equal weighting in the operation of a Delphi.[20 21] However, the Delphi method has been criticized as lacking objectivity and having problematic reliability, validity, and credibility.[22] This is because the outcomes from the Delphi method are solely based on experts` opinions, rather than on the more objective evidence from research studies like RCTs.

In the study, we will invite experts in the field defined as authors of the included reviews. We will invite one author per included review, or two if the number of reviews is too small. We attempt to include a total of 10-15 experts. If the number of reviews is greater than 15, we will draw a random sample. We will set up an initial video conference to clarify the aim of the consensus study. For the consensus study, we will ask the experts to rate the seriousness of the impact of spin patterns on research and clinical practice using a 7-point rating scale in which “1” indicates that the spin pattern is not serious at all and “7” indicates that the spin pattern is extremely

For peer review only - http://bmjopen.bmj.com/site/about/guidelines.xhtml

serious. In addition, and most importantly, the experts will also provide a written justification for the rating. The mean scores will be calculated and the range of the scores will be presented for each spin pattern. A researcher from the core team will present the results (i.e. anonymous ratings, justifications, and mean scores) to the individual group members in written form, ask them to review the results, and possibly adjust their own judgments based on the summary. We will repeat this process until no further changes occur. The final version will provide, for each spin pattern, the individual ratings (to show the level of agreement), the mean rating, and a justification (if the ratings are heterogeneous, then we will provide explanation for the disagreement). It is not the aim that all members agree on a specific numerical rating and justification, i.e. disagreement regarding the importance of some pattern is a possible result of the study. If necessary, e.g., to discuss questions that are too complex for written communication, we may set up additional video conferences. If necessary, we may first develop a new classification for spin patterns together with the experts using similar methods (see the section "Potential development of a comprehensive classification scheme for spin patterns").

Ethics and dissemination

The present protocol has been approved by Academic Centre for Dentistry Amsterdam (ACTA) Ethics Review Committee (2020250). We will publish results in a peer-reviewed scientific journal.

Authors` contributions

NS and CMF drafted the first version of the protocol and subsequently incorporated the suggested revisions. MvdL, GvdH, SL, and SS commented and revised each section of the protocol.

Funding

This research received no specific grant from any funding agency in the public, commercial or not-for-profit sectors.

Competing interests

None declared

For peer review only - http://bmjopen.bmj.com/site/about/guidelines.xhtml

Acknowledgement

We gratefully acknowledged Mr. René H.J. Otten, from University Library, Vrije Universiteit Amsterdam, the Netherlands for his assistance in the modification of the search strategies.

Patient consent

Not applicable

Provenance and peer review

Not commissioned; externally peer review

Patient and public involvement

Patients or the public will not be involved in the design, or conduct, or reporting, or dissemination plans of our research.

References

1. Chiu K, Grundy Q, Bero L. "Spin" in published biomedical literature: A methodological systematic review. *PLoS Biol* 2017;15:e2002173.
2. Ochodo EA, de Haan MC, Reitsma JB, et al. Overinterpretation and misreporting of diagnostic accuracy studies: Evidence of "spin". *Radiology* 2013;267:581-8.
3. Caulfield T, Ogbogu U. The commercialization of university-based research: balancing risks and benefits. *BMC Med Ethics* 2015;16:70.
4. Yavchitz A, Ravaud P, Altman DG, et al. A new classification of spin in systematic reviews and meta-analyses was developed and ranked according to the severity. *J Clin Epidemiol* 2016;75:56-65.
5. Lazarus C, Haneef R, Ravaud P, et al. Classification and prevalence of spin in abstracts of non-randomized studies evaluating and intervention. *BMC Med Res Methodol* 2015;15:85
6. Boutron I, Dutton S, Ravaud P, et al. Reporting and interpretation of randomized controlled trials with statistically nonsignificant results for primary outcomes. *JAMA* 2010;303:2058-64.

For peer review only - http://bmjopen.bmj.com/site/about/guidelines.xhtml

7. Cooper CM, Gray HM, Ross AE, et al. Evaluation of spin in the abstracts of otolaryngology randomized controlled trials. *Laryngoscope* 2018;21:2036-40.

8. Kempf E, de Beyer JA, Cook J, et al. Overinterpretation and misreporting of prognostic factor studies in oncology: a systematic review. *Br J Cancer* 2018;119:1288-96.

9. Boutron I, Altman DG, Hopewell S, et al. Impact of spin in the abstracts of articles reporting results of randomized controlled trials in the field of cancer: the SPIIN randomized controlled trial. *J Clin Oncol* 2014;32:4120-6.

10. Bero L, Chiu K, Grundy Q. The SSSPIN study-spin in studies of spin: meta-research analysis. *BMJ* 2019;367:I6202.

11. Boutron I, Haneef R, Yavchitz A, et al. Three randomized controlled trials evaluating the impact of "spin" in health news stories reporting studies of pharmacologic treatments on patients`/caregivers` interpretation of treatment benefit. *BMC Med* 2019;17:105.

12. Shamseer L, Moher D, Clarke M, et al. Preferred reporting items for systematic review and meta-analysis protocols (PRISMA-P) 2015: elaboration and explanation. *BMJ* 2015;350:g7647.

13. Greenhalgh T, Peacock R. Effectiveness and efficiency of search methods in systematic reviews of complex evidence: audit of primary sources. *BMJ* 2005;331:1064-5.

14. Briscoe S, Bethel A, Rogers M. Conduct and reporting of citation searching in Cochrane systematic reviews: A cross-sectional study. *Res Synth Methods* 2020;11:169-80.

15. Snyder H. Literature review as a research methodology: An overview and guidelines. *J Bus Res* 2019;104:333-9.

16. Shea BJ, Reeves BC, Wells G, et al. AMSTAR 2: a critical appraisal tool for systematic reviews that include randomised or non-randomised studies of healthcare interventions, or both. *BMJ* 2017;358:j4008.

17. Pieper D, Pollock M, Fernandes RM, et al. Epidemiology and reporting characteristics of overviews of reviews of healthcare interventions published 2012-2016: Protocol for a systematic review. *Syst Rev* 2017;6:73.

18. Higgins JPT, Green S, eds. *Cochrane Handbook for Systematic Reviews of Interventions*. *Version 5.1.0. Updated,* 2011.

For peer review only - http://bmjopen.bmj.com/site/about/guidelines.xhtml

19. Dalkey N, Helmer O. An experimental application of the Delphi methods to the use of experts. Manage Sci 1963;9:458-67.

20. Vernon W. The Delphi technique: A review. *Int J Ther Rehabil* 2009;16:69-76.

21. Donohoe HM, Needham RD. Moving best practice forward: Delphi characteristics, advantages, potential problems, and solutions. *Int J Tour Res* 2009;11:415-37.

22. Keeney S, Hasson F, McKenna HP. A critical review of the Delphi technique as a research methodology for nursing. *Int J Nurs Stud* 2001;38:195-200.

For peer review only

Table 1 Search strategies in PubMed, EMBASE, and SCOPUS

**PubMed**	
#11	Search: #10 AND ("2010"[Date-Publication]: "3000"[Date-Publication])
#10	Search: #8 OR #9
#9	Search: #7 AND systematic[sb]
#8	Search: #7 AND review[Publication Type]
#7	Search: #3 OR #4 OR #5 OR #6
#6	Search: ("misinterpretation"[tiab] OR "overinterpretation"[tiab]) AND "result*"[tiab]
#5	Search: "distorted results"[tiab] OR "distorted reporting"[tiab] OR "distorted presentation"[tiab] OR "distorted interpretation"[tiab]
#4	Search: "reporting bias"[tiab] OR "interpretation bias"[tiab]
#3	Search: #1 AND #2
#2	Search: "Publication Bias"[Mesh] OR "report*"[tiab] OR "bias*"[tiab] OR "publish*"[tiab] OR "non-significant*"[tiab] OR "nonsignificant*"[tiab]
#1	Search: "Spin"[tiab]
**EMBASE**	
#11	#10 AND [2010-2020]/py
#10	#8 OR #9
#9	#7 AND 'systematic review*':ti,ab,kw
#8	#7 AND [review]/lim
#7	#3 OR #4 OR #5 OR #6
#6	(('misinterpretation' OR 'overinterpretation') NEAR/4 'result*'):ti,ab,kw
#5	distorted results':ti,ab,kw OR 'distorted reporting':ti,ab,kw OR 'distorted presentation':ti,ab,kw OR 'distorted interpretation':ti,ab,kw
#4	'reporting bias'/exp OR 'interpretation bias'/exp
#3	#1 AND #2
#2	'publication bias'/exp OR 'report*':ti,ab,kw OR 'bias*':ti,ab,kw OR

For peer review only

	'publish*':ti,ab,kw OR 'non-significant*':ti,ab,kw OR 'nonsignificant*':ti,ab,kw
#1	'spin':ti,ab,kw
**SCOPUS**	
#10	#9 AND (PUBYEAR > 2009)
#9	#6 OR #8
#8	#4 AND #7
#7	TITLE-ABS-KEY ("systematic review*")
#6	#4 OR #5
#5	DOCTYPE (re)
#4	#1 OR #2 OR #3
#3	TITLE-ABS-KEY (("misinterpretation" OR "overinterpretation") W/3 ("result*"))
#2	TITLE-ABS-KEY ("reporting bias" OR "interpretation bias" OR "distorted results" OR "distorted reporting" OR "distorted presentation " OR "distorted interpretation")
#1	TITLE-ABS-KEY (("spin") W/3 ("report*" OR "bias*" OR "publish*" OR "non-significant*" OR "nonsignificant*"))

For peer review only

Table 2 The critical and non-critical items of AMSTAR-2 that are applicable in the present study

Items	Response options
Critical items	
Item 4 Did the review authors use a comprehensive literature search strategy?	Yes/Partial yes/No
Item 7 Did the review authors provide a list of excluded studies and justify the exclusions?	Yes/Partial yes/No
Item 13 Did the review authors account for RoB in individual studies when interpreting/discussing the results of the review?	Yes/No
Non-critical items	
Item 5 Did the review authors perform study selection in duplicate?	Yes/No
Item 6 Did the review authors perform data extraction in duplicate?	Yes/No
Item 8 Did the review authors describe the included studies in adequate detail?	Yes/Partial yes/No
Item 10 Did the review authors report on the sources of funding for the studies included in the review?	Yes/No
Item 16 Did the review authors report any potential sources of conflict of interest, including any funding they received for conducting the review?	Yes/No

For peer review only

For peer review only - http://bmjopen.bmj.com/site/about/guidelines.xhtml

PRISMA-P 2015 Checklist

This checklist has been adapted for use with protocol submissions to *Systematic Reviews* from Table 3 in Moher D et al: Preferred reporting items for systematic review and meta-analysis protocols (PRISMA-P) 2015 statement. *Systematic Reviews* 2015 **4**:1

Section/topic	#	Checklist item	Information reported: Yes	Information reported: No	Page number(s)
**ADMINISTRATIVE INFORMATION**					
**Title**					
Identification	1a	Identify the report as a protocol of a systematic review	✓	☐	1
Update	1b	If the protocol is for an update of a previous systematic review, identify as such	☐	☐	N.A.
**Registration**	2	If registered, provide the name of the registry (e.g., PROSPERO) and registration number in the Abstract	✓	☐	2
**Authors**					
Contact	3a	Provide name, institutional affiliation, and e-mail address of all protocol authors; provide physical mailing address of corresponding author	✓	☐	1
Contributions	3b	Describe contributions of protocol authors and identify the guarantor of the review	✓	☐	11
**Amendments**	4	If the protocol represents an amendment of a previously completed or published protocol, identify as such and list changes; otherwise, state plan for documenting important protocol amendments	☐	☐	N.A.
**Support**					
Sources	5a	Indicate sources of financial or other support for the review	✓	☐	11
Sponsor	5b	Provide name for the review funder and/or sponsor	☐	☐	N.A.
Role of sponsor/funder	5c	Describe roles of funder(s), sponsor(s), and/or institution(s), if any, in developing the protocol	☐	☐	N.A.
**INTRODUCTION**					
**Rationale**	6	Describe the rationale for the review in the context of what is already known	✓	☐	4-5
**Objectives**	7	Provide an explicit statement of the question(s) the review will address with reference to participants, interventions, comparators, and outcomes (PICO)	✓	☐	5

For peer review only - http://bmjopen.bmj.com/site/about/guidelines.xhtml

Section/topic	#	Checklist item	Information reported		Page number(s)
			Yes	No	
**METHODS**					
**Eligibility criteria**	8	Specify the study characteristics (e.g., PICO, study design, setting, time frame) and report characteristics (e.g., years considered, language, publication status) to be used as criteria for eligibility for the review	✓	☐	6-7
**Information sources**	9	Describe all intended information sources (e.g., electronic databases, contact with study authors, trial registers, or other grey literature sources) with planned dates of coverage	✓	☐	6
**Search strategy**	10	Present draft of search strategy to be used for at least one electronic database, including planned limits, such that it could be repeated	✓	☐	14-15 (Table 1)
STUDY RECORDS					
Data management	11a	Describe the mechanism(s) that will be used to manage records and data throughout the review	✓	☐	7
Selection process	11b	State the process that will be used for selecting studies (e.g., two independent reviewers) through each phase of the review (i.e., screening, eligibility, and inclusion in meta-analysis)	✓	☐	7-9
Data collection process	11c	Describe planned method of extracting data from reports (e.g., piloting forms, done independently, in duplicate), any processes for obtaining and confirming data from investigators	✓	☐	7
**Data items**	12	List and define all variables for which data will be sought (e.g., PICO items, funding sources), any pre-planned data assumptions and simplifications	✓	☐	7
**Outcomes and prioritization**	13	List and define all outcomes for which data will be sought, including prioritization of main and additional outcomes, with rationale	✓	☐	7
**Risk of bias in individual studies**	14	Describe anticipated methods for assessing risk of bias of individual studies, including whether this will be done at the outcome or study level, or both; state how this information will be used in data synthesis	✓	☐	7-8
DATA					
**Synthesis**	15a	Describe criteria under which study data will be quantitatively synthesized	✓	☐	10
	15b	If data are appropriate for quantitative synthesis, describe planned summary measures, methods of handling data, and methods of combining data from studies, including any planned exploration of consistency (e.g., I^2, Kendall's tau)	✓	☐	10
	15c	Describe any proposed additional analyses (e.g., sensitivity or subgroup analyses, meta-regression)	✓	☐	10
	15d	If quantitative synthesis is not appropriate, describe the type of summary planned	✓	☐	10
**Meta-bias(es)**	16	Specify any planned assessment of meta-bias(es) (e.g., publication bias across studies, selective reporting within studies)	✓	☐	10
**Confidence in**	17	Describe how the strength of the body of evidence will be assessed (e.g., GRADE)	☐	☐	N.A.

BioMed Central
The Open Access Publisher

Section/topic	#	Checklist item	Information reported		Page number(s)
			Yes	No	
**cumulative evidence**					

For peer review only

BMJ Open

Published patterns of spin in biomedical literature: A protocol for a meta-research study

Journal:	*BMJ Open*
Manuscript ID	bmjopen-2020-043784.R2
Article Type:	Protocol
Date Submitted by the Author:	19-Mar-2021
Complete List of Authors:	Su, Naichuan; Academic Centre for Dentistry Amsterdam, Department of Oral Public Health<br>van der Linden, Michiel; Academic Centre for Dentistry Amsterdam, Department of Oral Public Health<br>van der Heijden, Geert; Academic Centre for Dentistry Amsterdam, Department of Oral Public Health<br>Listl, Stefan; Radboud Universiteit, Department of Dentistry – Quality and Safety of Oral Healthcare, Radboud University Medical Center; Heidelberg University, Section for Translational Health Economics, Medical Faculty<br>Schandelmaier, Stefan; McMaster University, Department of Health Research Methods, Evidence, and Impact; University Hospital and University of Basel, Intitute for Clinical Epidemiology and Biostatistics, Department of Clinical Research<br>Faggion, Clovis M.; University Hospital Munster, Department of Periodontology and Operative Dentistry, Faculty of Dentistry
**Primary Subject Heading**:	Ethics
Secondary Subject Heading:	Epidemiology, Evidence based practice, Health policy
Keywords:	ETHICS (see Medical Ethics), EPIDEMIOLOGY, MEDICAL ETHICS

SCHOLARONE™
Manuscripts

I, the Submitting Author has the right to grant and does grant on behalf of all authors of the Work (as defined in the below author licence), an exclusive licence and/or a non-exclusive licence for contributions from authors who are: i) UK Crown employees; ii) where BMJ has agreed a CC-BY licence shall apply, and/or iii) in accordance with the terms applicable for US Federal Government officers or employees acting as part of their official duties; on a worldwide, perpetual, irrevocable, royalty-free basis to BMJ Publishing Group Ltd ("BMJ") its licensees and where the relevant Journal is co-owned by BMJ to the co-owners of the Journal, to publish the Work in this journal and any other BMJ products and to exploit all rights, as set out in our licence.

The Submitting Author accepts and understands that any supply made under these terms is made by BMJ to the Submitting Author unless you are acting as an employee on behalf of your employer or a postgraduate student of an affiliated institution which is paying any applicable article publishing charge ("APC") for Open Access articles. Where the Submitting Author wishes to make the Work available on an Open Access basis (and intends to pay the relevant APC), the terms of reuse of such Open Access shall be governed by a Creative Commons licence – details of these licences and which Creative Commons licence will apply to this Work are set out in our licence referred to above.

Other than as permitted in any relevant BMJ Author's Self Archiving Policies, I confirm this Work has not been accepted for publication elsewhere, is not being considered for publication elsewhere and does not duplicate material already published. I confirm all authors consent to publication of this Work and authorise the granting of this licence.

For peer review only

Published patterns of spin in biomedical literature: A protocol for a meta-research study

Naichuan Su,[1] Michiel van der Linden,[1] Geert van der Heijden,[1] Stefan Listl,[2,3] Stefan Schandelmaier,[4,5] Clovis Mariano Faggion Jr[6]

Author affiliations

[1]Department of Oral Public Health, Academic Centre for Dentistry Amsterdam (ACTA), University of Amsterdam and Vrije Universiteit Amsterdam, Amsterdam, the Netherlands

[2]Department of Dentistry – Quality and Safety of Oral Healthcare, Radboud University Medical Center, Radboud University, Nijmegen, the Netherlands

[3]Section for Translational Health Economics, Medical Faculty, Heidelberg University, Heidelberg, Germany

[4]Institute for Clinical Epidemiology and Biostatistics, Department of Clinical Research, University Hospital and University of Basel, Basel, Switzerland

[5]Department of Health Research Methods, Evidence, and Impact, McMaster University, Hamilton, Ontario, Canada

[6]Department of Periodontology and Operative Dentistry, Faculty of Dentistry, University Hospital Münster, Münster, Germany

Corresponding author:

Naichuan Su, Department of Oral Public Health, Academic Centre for Dentistry Amsterdam (ACTA), University of Amsterdam and Vrije Universiteit Amsterdam, Gustav Mahlerlaan 3004, 1081 LA Amsterdam, the Netherlands.

Email: n.su@acta.nl

Keywords: Spin; reporting bias; misinterpretation; prevalence

Word count: 4348

For peer review only - http://bmjopen.bmj.com/site/about/guidelines.xhtml

Abstract

Introduction Spin is defined as reporting practices that distort the interpretation of results and create misleading conclusions by suggesting more favorable results. Such unjustifiable and misleading misrepresentation may negatively influence the development of further studies, clinical practice, and healthcare policies. Spin manifests in various patterns in different sections of publications (titles, abstracts, and main texts). The primary aim of the present study is to identify reported spin patterns and assess the prevalence of spin in general and the prevalence of spin patterns reported in biomedical literature based on previously published systematic reviews and literature reviews on spin.

Methods and analysis PubMed, EMBASE, and SCOPUS will be searched to identify systematic or literature reviews on spin in biomedicine. To improve the comprehensiveness of the search, the snowballing method will be used to broaden the search. The data on spin-related outcomes and characteristics of the included studies will be extracted. The methodological quality of the included studies will be assessed with selective items of the AMSTAR-2 checklist. A new classification scheme for spin patterns will be developed if the classifications of spin patterns identified in the included studies vary. The prevalence of spin and spin patterns will be pooled based on meta-analyses if the classification schemes for spin are comparable across included studies. Otherwise, the prevalence will be described qualitatively. The seriousness of spin patterns will be assessed based on a Delphi consensus study.

Ethics and dissemination This study has been approved by the Academic Centre for Dentistry Amsterdam (ACTA) Ethics Review Committee (2020250). The study will be submitted to a peer-reviewed scientific journal.

Registration Open Science Framework (OSF): osf.io/hzv6e

For peer review only

Strength and limitations of this study

- While previous systematic or literature reviews on spin concerned the identification of spin patterns in various study designs, this will be the first systematic review focusing on the prevalence of spin patterns and seriousness of the impact of spin patterns on research and clinical practice based on the previously published systematic or literature reviews.
- This study may give insight into which spin patterns occur most frequently and may cause most serious consequences in research and clinical practice, and thus should be given most attention when researchers, clinicians, and policymakers read and write scientific papers.
- Potential limitation may be that the various definitions of spin and the various classification schemes for spin patterns used in the included studies may impact the pooling and interpretation of the results in the present study.
- Limiting the search to literature reviews or systematic reviews only is a potential limitation of the study because some meta-studies may not be reviews.
- Applying the AMSTAR-2 checklist to all the included studies for methodological quality assessment is another potential limitation because some of the included studies may have other designs than systematic reviews.

For peer review only - http://bmjopen.bmj.com/site/about/guidelines.xhtml

Introduction

Spin is defined as intentionally or unintentionally inaccurate, unfair, or partial reporting practices that distort the interpretation of research and create misleading conclusions by suggesting more favorable results.[1] It can be hypothesized that such unjustifiable and misleading misrepresentation may flatter research and make it more attractive for publication and citation and consequently may receive unwarrantably higher impact scores.

Spin is reported in 57% of the published clinical trials on average based on a systematic review on spin.[1] Based on the hypothesis, spin may lead to overpromising and misleading information in transfer of knowledge, introduce misconceptions to researchers, clinicians, and policymakers, and stimulate ineffective or even harmful financial investments from funding organizations and health institutions. Therefore, spin has a negative impact on advancing healthcare practice and population health, adversely influences health policy planning, adds to research waste, decreases the reproducibility of research, hampers the progress of science, and reduces return-of-investment from research.[1-3]

Spin manifests in different sections of publications (titles, abstracts, and main texts) and in various patterns. For example, the title of a publication may claim a beneficial effect of an experimental intervention which is not supported by the reported findings, or the conclusion of a publication is not supported by the reported findings, or the provided recommendations for clinical practice are not in line with the study conclusion. Those spin patterns can be classified into three main forms: misleading reporting, misleading interpretation, and inappropriate extrapolation.[4 5] The misleading reporting was defined as incomplete or inadequate reporting of the methods, study analysis, study results, or any important information that could be misleading to the readers, such as the selective reporting of or overemphasis on statistically significant secondary outcomes but ignoring the statistically non-significant primary outcomes. The misleading interpretation was defined as an interpretation of the study results that could be misleading to the readers, such as the conclusion claiming equivalence or comparable effectiveness for non-significant results. The inappropriate extrapolation

For peer review only

was defined as an inappropriate generalization of study results, such as the conclusion claiming the recommendation to use the treatment in clinical practice in an observational study.[1 4]

Several studies on the prevalence of spin and the impact of spin on research in general have been published. For example, multiple systematic reviews or literature reviews investigated spin in primary studies such as randomized controlled trials (RCTs), non-randomized controlled trials, diagnostic accuracy studies, prognostic studies, or systematic reviews performed in different clinical areas.[2 5-8] In addition, a meta-research study on spin was published in 2019, which aimed to identify spin and patterns of spin in the studies on spin.[9] Almost all these previous studies on spin showed that spin was very common across different types of the primary studies, ironically even in the studies on spin.[9] In addition, a study reported that spin in abstracts may influence clinicians` decision-making on the treatment effect in the field of cancer,[10] while another study reported that spin in health news stories reporting studies of pharmacologic treatment may influence both patients` and caregivers` decision-making on the treatment effect.[11] Another meta-research study on spin was published in 2016.[1] It focused on the theory of spin and the overall prevalence of spin in primary studies based on systematic or literature reviews on spin. These studies have revealed that spin is highly frequent in medical publications and can significantly impact both clinicians` and patients` decision-making in clinical practice. The main aim of these studies was to define, describe and understand spin. From the publications on spin available to date, different patterns of spin seem to emerge, but a comprehensive overview of spin patterns is lacking. Spin patterns vary in the prevalence in research and in the consequences on research. The ignorance on those spin patterns with the highest prevalence and the most serious impact on research may widely and severely harm the reliability, transparency, and accuracy of translation, dissemination, and implementation of the evidence from medical and health research to practice. Identification of the prevalence and the impact of spin patterns will help increase the awareness of researchers, clinicians, reviewers, and policymakers on which spin patterns should be given most attention when they read, review and write scientific papers. Besides, to develop the practical and targeted guidance on the identification and prevention of spin in research, the manifestations of spin should be known. The specific

For peer review only - http://bmjopen.bmj.com/site/about/guidelines.xhtml

and targeted guidance on how to identify and prevent such spin patterns in research can be made accordingly to help prevent spin in the future publications and prevent the use and transfer of the spinned evidence in research. To date, however, meta-research studies on the prevalence of spin patterns in biomedical publications and seriousness of the impact of different spin patterns on research and clinical practice are scarce.

Therefore, the aims of the study are to (1) identify reported spin patterns and assess the prevalence of spin in general and the prevalence of spin patterns reported in biomedical literature based on previously published systematic reviews and literature reviews on spin; and (2) assess the seriousness of the impact of spin patterns on research and clinical practice. Based on the spin patterns found in the current study which are most frequent in research or have the most serious consequences on research, we will derive recommendations for researchers, clinicians, and policymakers to prevent such spin patterns in future research.

Methods and analysis

The present protocol has been registered in the Open Science Framework (OSF) and reported in accordance with the Preferred Reporting Items for Systematic Review and Meta-analysis Protocols (PRISMA-P) statement.[12] The present protocol has been approved by Academic Centre for Dentistry Amsterdam (ACTA) Ethics Review Committee (2020250).

Search strategy

Relevant publications will be searched in electronic bibliographic sources, including PUBMED, EMBASE, and SCOPUS. The full search strategies, which have been modified by a senior librarian from Vrije Universiteit Amsterdam, the Netherlands (see acknowledgment), are described in Table 1. Searches will be limited from the last 10 years (from 2010), as from the first publication to identify spin in clinical trials.[6] The searching for the abstracts is planned to be done in early May 2021.

To improve the comprehensiveness of the search and identify potentially eligible studies which are not retrieved from the databases, the snowballing method will be used

For peer review only - http://bmjopen.bmj.com/site/about/guidelines.xhtml

to broaden the search.[13] That is, we inspect the references that are cited in the studies eligible for inclusion (backward citation searching).[14] Then, we use "see all similar articles" function at PubMed to check the first 20 most "similar studies" for each eligible publication retrieved from either the databases or the lists of references of each eligible publication.

Eligibility criteria

We will include publications that satisfy all of the following criteria:

1. They reported systematic or literature reviews on spin. That is, the publications aimed to examine spin for the primary studies in a specific or broad field of medicine. The review articles are defined as a more or less systematic way of collecting and synthesizing previous research.[15] A review will be defined as systematic when authors of the review made clear the intention of performing a systematic review. If a publication met the definition based on its adopted methods without using the word "review" in the titles, abstracts, or main texts, it will also be included.
2. They assessed spin of primary studies with any study design (e.g. effect of treatment or prevention studies (including randomized and non-randomised trials) and diagnostic and prognostic accuracy studies, economic studies and systematic reviews) in any field of biomedical sciences;
3. They reported possible spin patterns and prevalence of spin or of spin patterns in the included primary studies.
4. They were published in the English language.

Two reviewers (NS and CMF) will select a sample (10%) of eligible studies to achieve good agreement (at least 80%) on in- and exclusion of publications, and thereafter the remainder selected by one reviewer (NS).[16] Full texts will be obtained for studies that meet the inclusion criteria, and whenever title and abstract provide insufficient information for inclusion.

Data extraction

The characteristics of the included studies and spin-related outcomes will be extracted from each review directly to a standardized form. The standardized form will be

For peer review only - http://bmjopen.bmj.com/site/about/guidelines.xhtml

developed in Microsoft Excel software.

The information on the characteristics of the included studies include a) publication year and first authors; b) country/continent of the first author; c) number of authors of the review; d) type of review (systematic vs. literature review); e) medical specialty; f) type of primary study evaluated in the review; g) sponsorship of the review; h) conflict of interest of authors of the review; i) sampling methods used in the reviews.

The spin-related outcomes of the included studies include a) in what sections the spin was assessed (e.g. abstracts, methods, results, and conclusions sections of the primary studies); b) spin patterns identified in the reviews including labels, definitions, and classification schemes; c) prevalence of spin (overall and for different spin patterns) based on their original classification schemes.

Two reviewers (NS and CMF) will extract the data from a sample (10%) of eligible studies and achieve good agreement (at least 80%), with the remainder extracted by one reviewer (NS).[16]

Assessment of methodological quality

The updated version of A MeaSurement Tool to Assess systematic Reviews (AMSTAR-2), which includes a total of 16 items, is mainly used to assess the methodological quality of systematic reviews that include randomized or non-randomized studies of healthcare interventions.[16] Because there is no validated tool to evaluate the methodological systematic or literature reviews so far, we will apply selective items of the AMSTAR-2 checklist[16] that fit the purpose of our research to assess the methodological quality of the included systematic or literature review on spin. Eight items from the AMSTAR-2 items are applicable and selected in the present study (Table 2).

The response options to each item include "Yes" and "No". For some items, "Partial yes" is an additional response option.[16] If an included study fully adheres to the item, the answer to the item is judged as "Yes" and this indicates the study has a good methodological quality in the aspect of such item. If an included study partially adheres

For peer review only - http://bmjopen.bmj.com/site/about/guidelines.xhtml

to the item, the answer to the item is judged as "Partial yes". If an included study does not adhere to the item or provides no information to rate the item, the answer to the item is judged as "No".[16] We will not combine the items to create an overall score. Instead, the potential impact of each item on the results of the present study will be considered separately.

Two reviewers (NS and CMF) will evaluate the methodological quality of reviews from a sample (10%) of eligible reviews and achieve good agreement (at least 80%), with the remainder extracted by one reviewer (NS).[16]

Reviewers´ training and double-checking for accuracy

The two assessors (NS and CMF) will pilot the forms for the data extraction and the methodological quality assessment using a set of 5% of the included reviews.[17] After the piloting round, the results will be discussed and, if necessary, the forms will be refined.

A third reviewer (MvdL) will independently check a random sample (10%) for the different study´s phases (selection, extraction, and quality assessment of the data) for accuracy. Any remaining disagreements will be discussed among all authors to reach a consensus decision.

Statistical analysis

The overall prevalence of spin and the prevalence of each spin pattern presented in the included systematic or literature reviews will be described. We anticipate that the spin patterns identified in the included studies will vary with respect to their labels, definitions, and classification systems used to organize and present them. If the classifications of patterns of spin (and consequently overall spin which is the sum of different patterns) vary only slightly across reviews, we will pool the reported overall prevalences and the prevalences of each spin pattern separately across reviews based on meta-analysis for single proportions with random effect models. If, however, the categories of spin patterns (and thus the definitions of overall spin) vary substantially across reviews, the overall prevalence of spin and the prevalence of spin patterns will be described qualitatively. When the meta-analysis is possibly performed, statistical

For peer review only - http://bmjopen.bmj.com/site/about/guidelines.xhtml

heterogeneity of the meta-analysis will be assessed with I^2 test.[18] The heterogeneity is considered large if $I^2 > 50\%$. If it shows large heterogeneity and the number of included studies in a meta-analysis is >10, multivariate meta-regression analysis will be used to explore the possible factors for the heterogeneity. To assess the publication bias of the included studies, a funnel plot will be used when the number of the studies included in the meta-analysis is larger than 10.[18] The statistical analysis will be performed via R software 3.3 ((R Development Core Team).

Assessment of the seriousness of the impact of spin patterns

The seriousness of the impact of spin patterns indicates how much consequence a certain spin pattern may cause in research and clinical practice if such spin pattern occurs in biomedical publications. We are planning to perform a Delphi consensus study to rate the seriousness of the spin patterns. The Delphi method is a structured process which uses a series of questionnaires or rounds to gather and to provide information in a panel of experts.[19] Compared with other possible study designs (e.g. RCTs), the Delphi method is more simple to design and more flexible to conduct.[20] Besides, it enables anonymity, which encourages experts` creativity, honesty, independent thinking, and balanced consideration of ideas while reducing the risk of group dynamics negatively affecting the outcomes.[20 21] The experts` opinions are given equal weighting in the operation of a Delphi.[20 21] However, the Delphi method has been criticized as lacking objectivity and having problematic reliability, validity, and credibility.[22] This is because the outcomes from the Delphi method are solely based on experts` opinions, rather than on the more objective evidence from research studies like RCTs.

In the study, we will invite experts in the field defined as authors of the included reviews. We will invite one author per included review, or two if the number of reviews is too small. We attempt to include a total of 10-15 experts. If the number of reviews is greater than 15, we will draw a random sample. We will set up an initial video conference to clarify the aim of the consensus study. For the consensus study, we will ask the experts to rate the seriousness of the impact of spin patterns on research and clinical practice using a 7-point rating scale in which "1" indicates that the spin pattern is not serious at all and "7" indicates that the spin pattern is extremely serious. In addition, and most

For peer review only - http://bmjopen.bmj.com/site/about/guidelines.xhtml

importantly, the experts will also provide a written justification for the rating. The mean scores will be calculated and the range of the scores will be presented for each spin pattern. A researcher from the core team will present the results (i.e. anonymous ratings, justifications, and mean scores) to the individual group members in written form, ask them to review the results, and possibly adjust their own judgments based on the summary. We will repeat this process until no further changes occur. The final version will provide, for each spin pattern, the individual ratings (to show the level of agreement), the mean rating, and a justification (if the ratings are heterogeneous, then we will provide explanation for the disagreement). It is not the aim that all members agree on a specific numerical rating and justification, i.e. disagreement regarding the importance of some pattern is a possible result of the study. If necessary, e.g., to discuss questions that are too complex for written communication, we may set up additional video conferences. If necessary, we may first develop a new classification for spin patterns together with the experts using similar methods (see the section "Potential development of a comprehensive classification scheme for spin patterns").

Ethics and dissemination

The present protocol has been approved by Academic Centre for Dentistry Amsterdam (ACTA) Ethics Review Committee (2020250). We will publish results in a peer-reviewed scientific journal.

Authors` contributions

NS and CMF drafted the first version of the protocol and subsequently incorporated the suggested revisions. MvdL, GvdH, SL, and SS commented and revised each section of the protocol.

Funding

This research received no specific grant from any funding agency in the public, commercial or not-for-profit sectors.

Competing interests

None declared

For peer review only - http://bmjopen.bmj.com/site/about/guidelines.xhtml

Acknowledgement

We gratefully acknowledged Mr. René H.J. Otten, from University Library, Vrije Universiteit Amsterdam, the Netherlands for his assistance in the modification of the search strategies.

Patient consent

Not applicable

Provenance and peer review

Not commissioned; externally peer review

Patient and public involvement

Patients or the public will not be involved in the design, or conduct, or reporting, or dissemination plans of our research.

References

1. Chiu K, Grundy Q, Bero L. "Spin" in published biomedical literature: A methodological systematic review. *PLoS Biol* 2017;15:e2002173.
2. Ochodo EA, de Haan MC, Reitsma JB, et al. Overinterpretation and misreporting of diagnostic accuracy studies: Evidence of "spin". *Radiology* 2013;267:581-8.
3. Caulfield T, Ogbogu U. The commercialization of university-based research: balancing risks and benefits. *BMC Med Ethics* 2015;16:70.
4. Yavchitz A, Ravaud P, Altman DG, et al. A new classification of spin in systematic reviews and meta-analyses was developed and ranked according to the severity. *J Clin Epidemiol* 2016;75:56-65.
5. Lazarus C, Haneef R, Ravaud P, et al. Classification and prevalence of spin in abstracts of non-randomized studies evaluating and intervention. *BMC Med Res Methodol* 2015;15:85
6. Boutron I, Dutton S, Ravaud P, et al. Reporting and interpretation of randomized controlled trials with statistically nonsignificant results for primary outcomes. *JAMA* 2010;303:2058-64.

For peer review only - http://bmjopen.bmj.com/site/about/guidelines.xhtml

7. Cooper CM, Gray HM, Ross AE, et al. Evaluation of spin in the abstracts of otolaryngology randomized controlled trials. *Laryngoscope* 2018;21:2036-40.
8. Kempf E, de Beyer JA, Cook J, et al. Overinterpretation and misreporting of prognostic factor studies in oncology: a systematic review. *Br J Cancer* 2018;119:1288-96.
9. Bero L, Chiu K, Grundy Q. The SSSPIN study-spin in studies of spin: meta-research analysis. *BMJ* 2019;367:I6202.
10. Boutron I, Altman DG, Hopewell S, et al. Impact of spin in the abstracts of articles reporting results of randomized controlled trials in the field of cancer: the SPIIN randomized controlled trial. *J Clin Oncol* 2014;32:4120-6.
11. Boutron I, Haneef R, Yavchitz A, et al. Three randomized controlled trials evaluating the impact of "spin" in health news stories reporting studies of pharmacologic treatments on patients`/caregivers` interpretation of treatment benefit. *BMC Med* 2019;17:105.
12. Shamseer L, Moher D, Clarke M, et al. Preferred reporting items for systematic review and meta-analysis protocols (PRISMA-P) 2015: elaboration and explanation. *BMJ* 2015;350:g7647.
13. Greenhalgh T, Peacock R. Effectiveness and efficiency of search methods in systematic reviews of complex evidence: audit of primary sources. *BMJ* 2005;331:1064-5.
14. Briscoe S, Bethel A, Rogers M. Conduct and reporting of citation searching in Cochrane systematic reviews: A cross-sectional study. *Res Synth Methods* 2020;11:169-80.
15. Snyder H. Literature review as a research methodology: An overview and guidelines. *J Bus Res* 2019;104:333-9.
16. Shea BJ, Reeves BC, Wells G, et al. AMSTAR 2: a critical appraisal tool for systematic reviews that include randomised or non-randomised studies of healthcare interventions, or both. *BMJ* 2017;358:j4008.
17. Pieper D, Pollock M, Fernandes RM, et al. Epidemiology and reporting characteristics of overviews of reviews of healthcare interventions published 2012-2016: Protocol for a systematic review. *Syst Rev* 2017;6:73.
18. Higgins JPT, Green S, eds. *Cochrane Handbook for Systematic Reviews of Interventions*. *Version 5.1.0. Updated,* 2011.

For peer review only - http://bmjopen.bmj.com/site/about/guidelines.xhtml

19. Dalkey N, Helmer O. An experimental application of the Delphi methods to the use of experts. Manage Sci 1963;9:458-67.

20. Vernon W. The Delphi technique: A review. *Int J Ther Rehabil* 2009;16:69-76.

21. Donohoe HM, Needham RD. Moving best practice forward: Delphi characteristics, advantages, potential problems, and solutions. *Int J Tour Res* 2009;11:415-37.

22. Keeney S, Hasson F, McKenna HP. A critical review of the Delphi technique as a research methodology for nursing. *Int J Nurs Stud* 2001;38:195-200.

For peer review only

For peer review only - http://bmjopen.bmj.com/site/about/guidelines.xhtml

Table 1 Search strategies in PubMed, EMBASE, and SCOPUS

**PubMed**	
#11	Search: #10 AND ("2010"[Date-Publication]: "3000"[Date-Publication])
#10	Search: #8 OR #9
#9	Search: #7 AND systematic[sb]
#8	Search: #7 AND review[Publication Type]
#7	Search: #3 OR #4 OR #5 OR #6
#6	Search: ("misinterpretation"[tiab] OR "overinterpretation"[tiab]) AND "result*"[tiab]
#5	Search: "distorted results"[tiab] OR "distorted reporting"[tiab] OR "distorted presentation"[tiab] OR "distorted interpretation"[tiab]
#4	Search: "reporting bias"[tiab] OR "interpretation bias"[tiab]
#3	Search: #1 AND #2
#2	Search: "Publication Bias"[Mesh] OR "report*"[tiab] OR "bias*"[tiab] OR "publish*"[tiab] OR "non-significant*"[tiab] OR "nonsignificant*"[tiab]
#1	Search: "Spin"[tiab]
**EMBASE**	
#11	#10 AND [2010-2020]/py
#10	#8 OR #9
#9	#7 AND 'systematic review*':ti,ab,kw
#8	#7 AND [review]/lim
#7	#3 OR #4 OR #5 OR #6
#6	(('misinterpretation' OR 'overinterpretation') NEAR/4 'result*'):ti,ab,kw
#5	distorted results':ti,ab,kw OR 'distorted reporting':ti,ab,kw OR 'distorted presentation':ti,ab,kw OR 'distorted interpretation':ti,ab,kw
#4	'reporting bias'/exp OR 'interpretation bias'/exp
#3	#1 AND #2
#2	'publication bias'/exp OR 'report*':ti,ab,kw OR 'bias*':ti,ab,kw OR 'publish*':ti,ab,kw OR 'non-significant*':ti,ab,kw OR

For peer review only

For peer review only - http://bmjopen.bmj.com/site/about/guidelines.xhtml

	'nonsignificant*':ti,ab,kw
#1	'spin':ti,ab,kw
**SCOPUS**	
#10	#9 AND (PUBYEAR > 2009)
#9	#6 OR #8
#8	#4 AND #7
#7	TITLE-ABS-KEY ("systematic review*")
#6	#4 OR #5
#5	DOCTYPE (re)
#4	#1 OR #2 OR #3
#3	TITLE-ABS-KEY (("misinterpretation" OR "overinterpretation") W/3 ("result*"))
#2	TITLE-ABS-KEY ("reporting bias" OR "interpretation bias" OR "distorted results" OR "distorted reporting" OR "distorted presentation " OR "distorted interpretation")
#1	TITLE-ABS-KEY (("spin") W/3 ("report*" OR "bias*" OR "publish*" OR "non-significant*" OR "nonsignificant*"))

For peer review only

For peer review only - http://bmjopen.bmj.com/site/about/guidelines.xhtml

1

2 **Table 2 The critical and non-critical items of AMSTAR-2 that are applicable in**

3 **the present study**

Items	Response options
Critical items	
Item 4 Did the review authors use a comprehensive literature search strategy?	Yes/Partial yes/No
Item 7 Did the review authors provide a list of excluded studies and justify the exclusions?	Yes/Partial yes/No
Item 13 Did the review authors account for RoB in individual studies when interpreting/discussing the results of the review?	Yes/No
Non-critical items	
Item 5 Did the review authors perform study selection in duplicate?	Yes/No
Item 6 Did the review authors perform data extraction in duplicate?	Yes/No
Item 8 Did the review authors describe the included studies in adequate detail?	Yes/Partial yes/No
Item 10 Did the review authors report on the sources of funding for the studies included in the review?	Yes/No
Item 16 Did the review authors report any potential sources of conflict of interest, including any funding they received for conducting the review?	Yes/No

4

For peer review only - http://bmjopen.bmj.com/site/about/guidelines.xhtml

PRISMA-P 2015 Checklist

This checklist has been adapted for use with protocol submissions to *Systematic Reviews* from Table 3 in Moher D et al: Preferred reporting items for systematic review and meta-analysis protocols (PRISMA-P) 2015 statement. *Systematic Reviews* 2015 **4**:1

Section/topic	#	Checklist item	Information reported Yes	Information reported No	Page number(s)
**ADMINISTRATIVE INFORMATION**					
**Title**					
Identification	1a	Identify the report as a protocol of a systematic review	✓	☐	1
Update	1b	If the protocol is for an update of a previous systematic review, identify as such	☐	☐	N.A.
**Registration**	2	If registered, provide the name of the registry (e.g., PROSPERO) and registration number in the Abstract	✓	☐	2
**Authors**					
Contact	3a	Provide name, institutional affiliation, and e-mail address of all protocol authors; provide physical mailing address of corresponding author	✓	☐	1
Contributions	3b	Describe contributions of protocol authors and identify the guarantor of the review	✓	☐	11
**Amendments**	4	If the protocol represents an amendment of a previously completed or published protocol, identify as such and list changes; otherwise, state plan for documenting important protocol amendments	☐	☐	N.A.
**Support**					
Sources	5a	Indicate sources of financial or other support for the review	✓	☐	11
Sponsor	5b	Provide name for the review funder and/or sponsor	☐	☐	N.A.
Role of sponsor/funder	5c	Describe roles of funder(s), sponsor(s), and/or institution(s), if any, in developing the protocol	☐	☐	N.A.
**INTRODUCTION**					
**Rationale**	6	Describe the rationale for the review in the context of what is already known	✓	☐	4-5
**Objectives**	7	Provide an explicit statement of the question(s) the review will address with reference to participants, interventions, comparators, and outcomes (PICO)	✓	☐	5

For peer review only

BioMed Central The Open Access Publisher

Section/topic	#	Checklist item	Information reported		Page number(s)
			Yes	No	
**METHODS**					
**Eligibility criteria**	8	Specify the study characteristics (e.g., PICO, study design, setting, time frame) and report characteristics (e.g., years considered, language, publication status) to be used as criteria for eligibility for the review	✓	☐	6-7
**Information sources**	9	Describe all intended information sources (e.g., electronic databases, contact with study authors, trial registers, or other grey literature sources) with planned dates of coverage	✓	☐	6
**Search strategy**	10	Present draft of search strategy to be used for at least one electronic database, including planned limits, such that it could be repeated	✓	☐	14-15 (Table 1)
STUDY RECORDS					
Data management	11a	Describe the mechanism(s) that will be used to manage records and data throughout the review	✓	☐	7
Selection process	11b	State the process that will be used for selecting studies (e.g., two independent reviewers) through each phase of the review (i.e., screening, eligibility, and inclusion in meta-analysis)	✓	☐	7-9
Data collection process	11c	Describe planned method of extracting data from reports (e.g., piloting forms, done independently, in duplicate), any processes for obtaining and confirming data from investigators	✓	☐	7
**Data items**	12	List and define all variables for which data will be sought (e.g., PICO items, funding sources), any pre-planned data assumptions and simplifications	✓	☐	7
**Outcomes and prioritization**	13	List and define all outcomes for which data will be sought, including prioritization of main and additional outcomes, with rationale	✓	☐	7
**Risk of bias in individual studies**	14	Describe anticipated methods for assessing risk of bias of individual studies, including whether this will be done at the outcome or study level, or both; state how this information will be used in data synthesis	✓	☐	7-8
DATA					
**Synthesis**	15a	Describe criteria under which study data will be quantitatively synthesized	✓	☐	10
	15b	If data are appropriate for quantitative synthesis, describe planned summary measures, methods of handling data, and methods of combining data from studies, including any planned exploration of consistency (e.g., I^2, Kendall's tau)	✓	☐	10
	15c	Describe any proposed additional analyses (e.g., sensitivity or subgroup analyses, meta-regression)	✓	☐	10
	15d	If quantitative synthesis is not appropriate, describe the type of summary planned	✓	☐	10
**Meta-bias(es)**	16	Specify any planned assessment of meta-bias(es) (e.g., publication bias across studies, selective reporting within studies)	✓	☐	10
**Confidence in**	17	Describe how the strength of the body of evidence will be assessed (e.g., GRADE)	☐	☐	N.A.

BioMed Central The Open Access Publisher

Section/topic	#	Checklist item	Information reported		Page number(s)
			Yes	No	
**cumulative evidence**					

For peer review only

BioMed Central
The Open Access Publisher
